# Beta Test of a PAC Dementia Knowledge Training Module Included in a Nursing Home Orientation Curriculum

**DOI:** 10.1093/geroni/igaa057.290

**Published:** 2020-12-16

**Authors:** Jerry Brown, Peter Baylie, Candidus Nwakasi, Pranay Reddy, Katie Ehlman, Beth Nolan, Teepa Snow

**Affiliations:** 1 University of Southern Indiana, Evansville, Indiana, United States; 2 Positive Approach to Care, Ada, Michigan, United States; 3 Teepa Snow, Efland, North Carolina, United States

## Abstract

Providing quality care for older adults in long-term care can be challenging, and this issue appears to be more pressing with people living with dementia. Using the Positive Approach® to Care (PAC) model, a 2-hour module for new staff nursing home orientation curriculum was designed to help introduce the concept of working with people living with dementia. Twelve undergraduate students participated in a beta-test of the nursing home orientation. A pre-and-post 38-item survey was administered to measure knowledge level and improvement. Participants also responded to qualitative semi-structured questions after the orientation. Descriptive statistics and bivariate analysis were conducted. Results indicated an improvement on dementia-related knowledge in most of the survey items (21 of 34 items). Examples of statistically significant differences in the pretest and post-test identified are knowledge on the effect of pressure in the palm to comfort a person with dementia (p-0.039), vision as the most powerful sensory input during dementia caregiving (p-0.001), and functionalities lost when the left temporal lobe shrinks (p-0.014). The qualitative evaluation showed that most of the participants indicated a change in dementia caregiving views — including how to pause if permission is not given to engage, and to respect personal space. These findings prove important because the PAC orientation curriculum was successful in improving the students’ knowledge and perspectives on dementia. This training program could be a useful tool if implemented into nursing home employee orientation.

